# Clinical [^18^F]FSPG Positron Emission Tomography Imaging Reveals Heterogeneity in Tumor-Associated System x_c_^−^ Activity

**DOI:** 10.3390/cancers16071437

**Published:** 2024-04-08

**Authors:** Amy R. Sharkey, Norman Koglin, Erik S. Mittra, Sangwon Han, Gary J. R. Cook, Timothy H. Witney

**Affiliations:** 1 School of Biomedical Engineering and Imaging Sciences, King’s College London, London SE1 7EH, UK; amyrose.sharkey@gstt.nhs.uk (A.R.S.); gary.cook@kcl.ac.uk (G.J.R.C.); 2 Life Molecular Imaging, 13353 Berlin, Germany; n.koglin@life-mi.com; 3 Division of Molecular Imaging and Therapy, Oregon Health & Science University, Portland, OR 97239, USA; mittra@ohsu.edu; 4 Department of Nuclear Medicine, Asan Medical Center, University of Ulsan College of Medicine, Seoul 05505, Republic of Korea; hswon87@naver.com; 5 King’s College London and Guy’s and St. Thomas’ PET Center, St. Thomas’ Hospital, London SE1 7EH, UK

**Keywords:** [^18^F]FSPG, redox, NSCLC, HNSCC, heterogeneity

## Abstract

**Simple Summary:**

This clinical study explored the use of the positron emission tomography radiotracer, [^18^F]FSPG, for cancer imaging. [^18^F]FSPG measures a process involved in antioxidant production, which is important for cancer prognosis. We compared the distribution of this imaging agent in subjects with head and neck squamous cell cancer (HNSCC) and non-small-cell lung cancer (NSCLC). Results showed similar distribution in healthy organs but varied uptake in tumors, both between subjects and across different lesions. Although [^18^F]FSPG PET/CT offers insights into individual tumor behavior, its diagnostic potential is limited due to this variability in tumor uptake. Variability in [^18^F]FSPG retention, however, may provide crucial information about how tumors respond to therapy and mechanisms of treatment resistance.

**Abstract:**

Background: (4*S*)-4-(3-[^18^F]fluoropropyl)-L-glutamic acid ([^18^F]FSPG) positron emission tomography/computed tomography (PET/CT) provides a readout of system x_c_^−^ transport activity and has been used for cancer detection in clinical studies of different cancer types. As system x_c_^−^ provides the rate-limiting precursor for glutathione biosynthesis, an abundant antioxidant, [^18^F]FSPG imaging may additionally provide important prognostic information. Here, we performed an analysis of [^18^F]FSPG radiotracer distribution between primary tumors, metastases, and normal organs from cancer patients. We further assessed the heterogeneity of [^18^F]FSPG retention between cancer types, and between and within individuals. Methods: This retrospective analysis of prospectively collected data compared [^18^F]FSPG PET/CT in subjects with head and neck squamous cell cancer (HNSCC, *n* = 5) and non-small-cell lung cancer (NSCLC, *n* = 10), scanned at different institutions. Using semi-automated regions of interest drawn around tumors and metastases, the maximum standardized uptake value (SUV_max_), SUV_mean_, SUV standard deviation and SUV_peak_ were measured. [^18^F]FSPG time–activity curves (TACs) for normal organs, primary tumors and metastases were subsequently compared to ^18^F-2-fluoro-2-deoxy-D-glucose ([^18^F]FDG) PET/CT at 60 min post injection (p.i.). Results: The mean administered activity of [^18^F]FSPG was 309.3 ± 9.1 MBq in subjects with NSCLC and 285.1 ± 11.3 MBq in those with HNSCC. The biodistribution of [^18^F]FSPG in both cohorts showed similar TACs in healthy organs from cancer patients. There was no statistically significant overall difference in the average SUV_max_ of tumor lesions at 60 min p.i. for NSCLC (8.1 ± 7.1) compared to HNSCC (6.0 ± 4.1; *p* = 0.29) for [^18^F]FSPG. However, there was heterogeneous retention between and within cancer types; the SUV_max_ at 60 min p.i. ranged from 1.4 to 23.7 in NSCLC and 3.1–12.1 in HNSCC. Conclusion: [^18^F]FSPG PET/CT imaging from both NSCLC and HNSCC cohorts showed the same normal-tissue biodistribution, but marked tumor heterogeneity across subjects and between lesions. Despite rapid elimination through the urinary tract and low normal-background tissue retention, the diagnostic potential of [^18^F]FSPG was limited by variability in tumor retention. As [^18^F]FSPG retention is mediated by the tumor’s antioxidant capacity and response to oxidative stress, this heterogeneity may provide important insights into an individual tumor’s response or resistance to therapy.

## 1. Background

**Figure 1:**
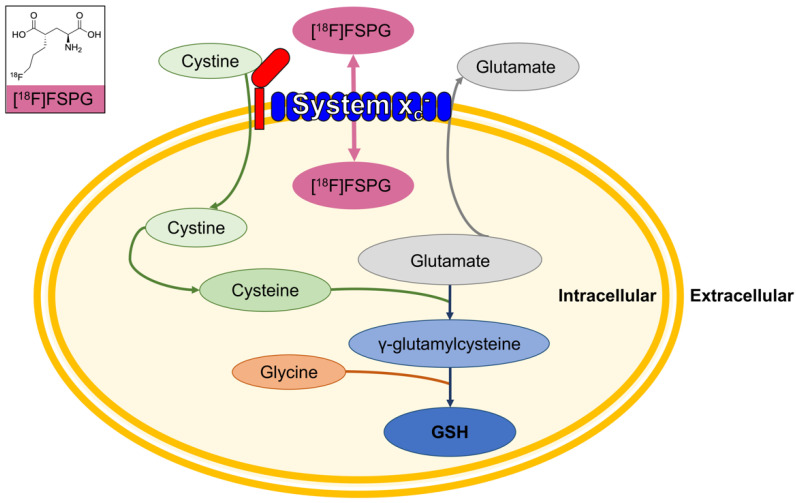
[^18^F]FSPG images system x_c_^−^ activity, providing a surrogate marker of glutathione (GSH) biosynthesis. Insert, the structure of [^18^F]FSPG.

Tumor cells require nutrients to meet their anabolic and energetic needs while maintaining an appropriate redox balance for growth, proliferation, and survival. Frequently, tumors experience high levels of oxidative stress following exposure to cellular oxidants, or conversely through the depletion of protective antioxidants. Tumors can adapt to this oxidative stress and maintain redox homeostasis by increasing antioxidant production [1], the most abundant of which is glutathione (GSH). The degree of oxidative stress within cells can be imaged and quantified using the positron emission tomography (PET) tracer (4*S*)-4-(3-[^18^F]fluoropropyl)-L-glutamic acid ([^18^F]FSPG), which is specifically transported by the cystine-glutamate antiporter system x_c_^−^ [2] (Figure 1). Net [^18^F]FSPG retention is a measure of the bidirectional transport across the cell membrane.

System x_c_^−^ is overexpressed in multiple tumor types [2,3], where it provides the rate-limiting precursor for GSH biosynthesis. Consequently, system x_c_^−^ activity (and its imaging with [^18^F]FSPG) provides a surrogate marker of the tumor’s antioxidant capacity, which has important implications for cancer progression and treatment resistance [4,5]. [^18^F]FSPG has been tested in humans, showing acceptable radiation dosimetry [6] and pharmacokinetic profile [7]. In healthy volunteers, [^18^F]FSPG was rapidly cleared from the blood pool by the kidneys and was retained at low levels in all healthy organs except the pancreas, which showed sustained retention [7]. 

Given the high expression of system x_c_^−^ across multiple cancer types, [^18^F]FSPG PET/CT has been evaluated for cancer detection, with a small number of published studies reporting high tumor-to-background contrast in lymphoma [8], brain [2,9], head and neck [8], breast [10], lung [10,11], liver [3,12], pancreatic [13], colorectal [8], and prostate cancers [14]. These studies compared the diagnostic performance of [^18^F]FSPG to standard-of-care imaging, most often ^18^F-2-fluoro-2-deoxy-D-glucose ([^18^F]FDG) PET/CT, reporting mixed results. In some patient populations [^18^F]FSPG produced favorable tumor-to-background ratios and high cancer detection rates, such as in hepatocellular carcinoma [3]. In other cancer types, for example breast cancer, diagnostic accuracy with [^18^F]FSPG was less favorable, with low tumor-to-background ratios and reduced tumor detection rates compared to [^18^F]FDG [12]. A summary of these studies and their implications for cancer patient management has recently been published [15].

Preclinical data, however, suggest that the clinical utility of [^18^F]FSPG lies not as a general diagnostic agent, but in the early monitoring of therapy response and prediction of treatment resistance. In animal models, tumor cell retention of [^18^F]FSPG was reduced in proportion to the levels of oxidative stress induced following treatment with redox-active compounds such as doxorubicin [16]. This decrease in [^18^F]FSPG retention was mediated by alterations in intracellular cystine (Figure 1), whose utilization is changed in response to oxidative stress [16]. Cystine is the rate-limiting precursor of glutathione. Resistance to cancer treatment, such as chemotherapy, is frequently a consequence of increased tumor glutathione biosynthesis and the resulting antioxidant capacity. Preclinically, [^18^F]FSPG retention was a sensitive marker of antioxidant production: in treatment-sensitive tumors prior to treatment, [^18^F]FSPG retention was high, whereas [^18^F]FSPG retention was low in treatment-resistant tumors [17]. It remains to be seen whether these findings translate to human studies.

Understanding therapy response with [^18^F]FSPG on a lesion-by-lesion basis may have important implications for patient outcomes. Most of the available clinical studies make qualitative statements regarding the heterogenous nature of [^18^F]FSPG retention, both between cancer types (inter-tumoral) and between the primary tumor and metastatic deposits (intra-subject); however, no quantitative analysis describing the extent of heterogeneity has been performed. Here, we aim to assess the inter-tumoral and inter-subject heterogeneity of [^18^F]FSPG retention via the retrospective analysis of prospective [^18^F]FSPG PET/CT imaging across two different cancer types: head and neck squamous cell cancer (HNSCC), and subjects with NSCLC. We selected these two cancer subtypes as they are cancers in which there is an urgent clinical need for new methods to assess treatment resistance and improve outcomes. Ultimately, we hypothesize that heterogeneity of [^18^F]FSPG retention is related to variation in underlying tumor redox homeostasis, and that variable uptake, as assessed here, could be clinically useful in terms of the prediction of treatment response and assessment of treatment resistance. 

## 2. Data and Methods

### 2.1. [^18^F]FSPG Acquisition

The anonymized [^18^F]FSPG PET/CT scans of ten subjects with NSCLC [10], imaged in South Korea, and five subjects with HNSCC [8], imaged in the USA, were analyzed. This study represents a retrospective analysis of prospectively collected imaging data originally reported by Baek et al. [10] and Park et al. [8]. Full methodological details and scanning protocols are described in the original papers with both sites undergoing regular standard PET quality control procedures. Baek et al. imaged patients using Siemens scanners (Biograph True Point 40 or Biograph 16; Siemens Healthineers, Erlangen, Germany). A low-dose CT (80 kV CARE Dose 4D, 31 mAs) was acquired with each PET scan. 

Park et al. imaged patients with GE Discovery PET/CT scanners (either model D600 or D690; GE HealthCare, Chicago, IL, USA). A low-dose CT (140 kV, range 10–85 mAs) was performed with each acquisition. PET scans at both sites were corrected for randoms, scatter, and attenuation using the CT image. Data were reconstructed using the manufacturer-provided ordered-subset expectation maximization algorithm. No correction for partial volume effects was conducted.

Both studies received local ethics approval. Approval for HNSCC subjects was granted from the U.S. Food and Drug Administration (eIND 108509), the Institutional Review Board at Stanford University, and the Scientific Review Committee at the Stanford Cancer Institute (IRB 15329). For NSCLC, approval was granted from the Institutional Review Board of the Asan Medical Center (University of Ulsan College of Medicine, Seoul, Republic of Korea) and the Korea Food and Drug Administration (IRB 2010-0054). All subjects provided written informed consent before participation in the study.

In the ten subjects with NSCLC, all subjects underwent [^18^F]FSPG imaging at approximately 0, 5, 10, 15, 30, 60 and 90 min p.i., and comparisons were made with [^18^F]FDG PET/CT at 60 min. In five subjects with HNSCC, four underwent [^18^F]FSPG imaging at approximately 0, 5, 10, 15, 30, 60 and 90 min p.i., and one underwent [^18^F]FSPG imaging at approximately 30, 60 and 90 min p.i. only. As for NSCLC subjects, comparisons were made with [^18^F]FDG PET/CT at 60 min. 

### 2.2. Image Analysis

A single radiologist (A.R.S), with 4 years’ experience, analyzed the imaging data. Primary lesions and metastases were identified on PET imaging and correlated with the CT images. Semi-automated spherical volumes between 1.5 cm and 3 cm in diameter were placed in normal organs (liver, pancreatic head and tail, spleen, renal cortices, blood pool at the aortic arch and bone marrow). In the primary tumor, and in all metastatic deposits >1 cm^3^, SUV_max_, SUV_mean_, SUV_peak_, SUV standard deviation, and metabolic tumor volume (MTV) values were acquired using a 40% maximum activity threshold using Hermes GOLD™ (Hermes Medical Solutions, Stockholm, Sweden). To minimize the effect of partial volume, we used SUV_max_ as our primary comparative metric. Time–activity curves (TAC) for normal organs, primary tumors and metastases were produced using Graphpad Prism for macOS version 9.5.1, GraphPad Software, San Diego, CA, USA.

### 2.3. Statistical Analysis

Differences in SUV_max_ across cohorts were measured using a two-tailed Student’s t-test as the data were normally distributed, with significance set at a *p*-value of 0.05. To evaluate intra-observer reliability, a second observer (G.J.R.C.), with 32 years’ experience, performed the analysis on 73 measurements in the HNSCC dataset and 170 measurements in the NSCLC dataset. The intra-class correlation coefficient was calculated using Microsoft Excel for macOS (version 6.73), and agreement was tested using Bland–Altman plots using Graphpad Prism. 

## 3. Results

### 3.1. Subject Demographics

Subject demographics are described in Table 1. In the HNSCC cohort, all subjects were male, with a mean age of 66.8 ± 6.7 years. Two of the subjects had progressive disease despite previous treatment for their HNSCC. In the NSCLC cohort, three subjects were female and seven were male, with a mean age of 59.7 ± 12.8 years. Of the NSCLC cohort, eight subjects had an adenocarcinoma and two had a squamous cell cancer. None had previously received treatment for their cancer. The mean administered activity of [^18^F]FSPG was 309.3 ± 9.1 MBq in NSCLC subjects and 285.1 ± 11.3 MBq in HNSCC subjects. 

### 3.2. [^18^F]FSPG Has Consistent Biodistribution in Normal Organs

**Figure 2:**
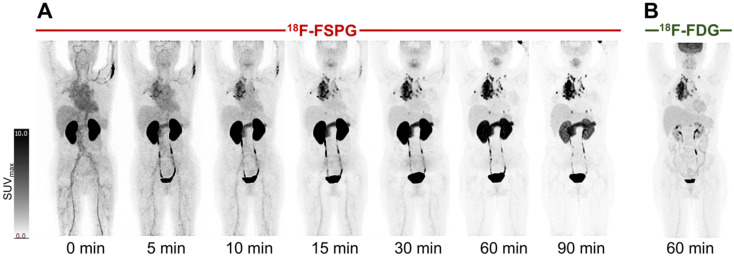
[^18^F]FSPG maximum intensity projection scans at intervals up to 90 min p.i. in a subject (subject 2) with metastatic lung cancer (**A**), demonstrating rapid accumulation in lung cancer and metastatic deposits, with an SUV_max_ of 23.7, compared to a standard 60 min p.i. [^18^F]FDG PET scan, with a lower SUV_max_ of 16.9 (**B**). Normal uptake in the oropharyngeal region relates to the soft palate.

**Figure 3:**
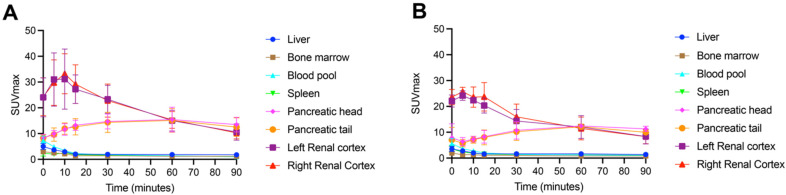
Time–activity curves of normal organ [^18^F]FSPG retention (average SUV_max_) in subjects with NSCLC (**A**) *n* = 10, acquired in South Korea) and HNSCC (**B**) *n* = 5, acquired in the USA).

Example [^18^F]FSPG PET/CT images from a subject with NSCLC are shown in Figure 2. As seen in all subjects over the seven time points (0–90 min), [^18^F]FSPG was rapidly cleared from the blood pool following injection, with similar rapid clearance from the liver, spleen and bone marrow. High [^18^F]FSPG retention was observed in the pancreas, which was sustained up to 90 min p.i., with a mean SUV_max_ of 10.6 (pancreatic head) and 9.4 (pancreatic tail) at 90 min p.i. Initial high renal cortical radioactivity peaked at 10 min (mean SUV_max_ of 24.6) before subsequently reducing due to urinary excretion, leaving only residual renal cortical activity after 60 min p.i. [^18^F]FSPG TACs in normal organs are shown in Figure 3, with SUV_max_ at each time point representing the average organ retention across subjects. [^18^F]FSPG displayed consistent uptake kinetics within organs in all subjects across both cohorts scanned at different centers.

### 3.3. [^18^F]FSPG Retention Is Heterogeneous in Both NSCLC and HNSCC Primary Tumors 

**Figure 4:**
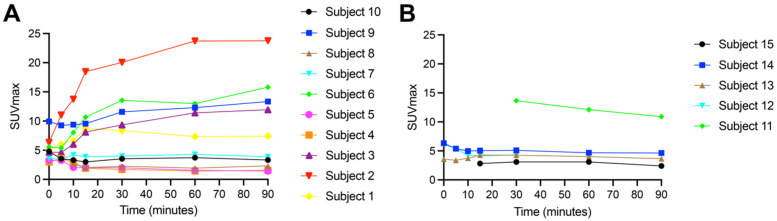
Retention of [^18^F]FSPG (average SUV_max_) in the primary tumor for subjects with NSCLC (**A**) *n* = 10 and HNSCC (**B**) *n* = 5. Numbered key correlates with subject number in Table 2.

The primary tumor retention of [^18^F]FSPG reached a plateau at approximately 60 min p.i. in both NSCLC and HNSCC (Figure 4). [^18^F]FSPG retention was higher in NSCLC (average SUV_max_ at 60 min p.i. for NSCLC primary tumors (8.1 ± 7.1) vs. HNSCC primary tumors (6.0 ± 4.1)), but this difference did not reach statistical significance due to the variability in retention (*p* = 0.29). Detailed data for the primary tumor uptake are shown in Table 2, which compares the SUV_max_, SUV_mean_, SUV_peak_, SUV standard deviation, and MTV values using [^18^F]FSPG vs. [^18^F]FDG at 60 min. In the primary tumor, the median long axis dimension was 27.5 mm (21–80 mm) in NSCLC, and 28 mm (20–48 mm) in HSNCC. Correlation plots of the SUV_max_ of [^18^F]FDG vs. [^18^F]FSPG are shown in Supplementary Figure S1, demonstrating a weakly positive but non-statistically significant correlation in NSCLC primary tumors and metastases, and no correlation in HNSCC primary tumors.

In subjects with NSCLC, there was measurable [^18^F]FSPG retention above background at all time points. Unlike the similar TACs seen with normal organs in both cohorts, the TACs for the treatment-naïve primary lesions showed marked heterogeneity in [^18^F]FSPG primary tumor retention between subjects, with the SUV_max_ at 60 min ranging from 1.4 to 23.7. Some subjects had primary tumors with very high [^18^F]FSPG accumulation, for example subject 2, who had rapid initial delivery of [^18^F]FSPG over the first 15 min p.i., reaching an SUV_max_ of 18.5, with radioactivity reaching a plateau between 60 (SUV_max_, 23.7) and 90 min (SUV_max_, 23.8; Figure 4, Table 2). Other subjects, i.e., subjects 4, 5 and 8, had low [^18^F]FSPG retention across all time points (Figure 4, Table 2), with SUV_max_ measurements of 1.4, 1.6 and 1.9 at 60 min p.i., respectively. Other subjects had intermediate retention, for example subjects 1 and 9, with SUV_max_ measurements of 7.3 and 12.3 at 60 min p.i., respectively. The subjects with a squamous cell subtype of NSCLC (3 and 6) both had intermediate [^18^F]FSPG retention, with SUV_max_ measurements of 11.4 and 13.0 at 60 min p.i., respectively. 

**Figure 5:**
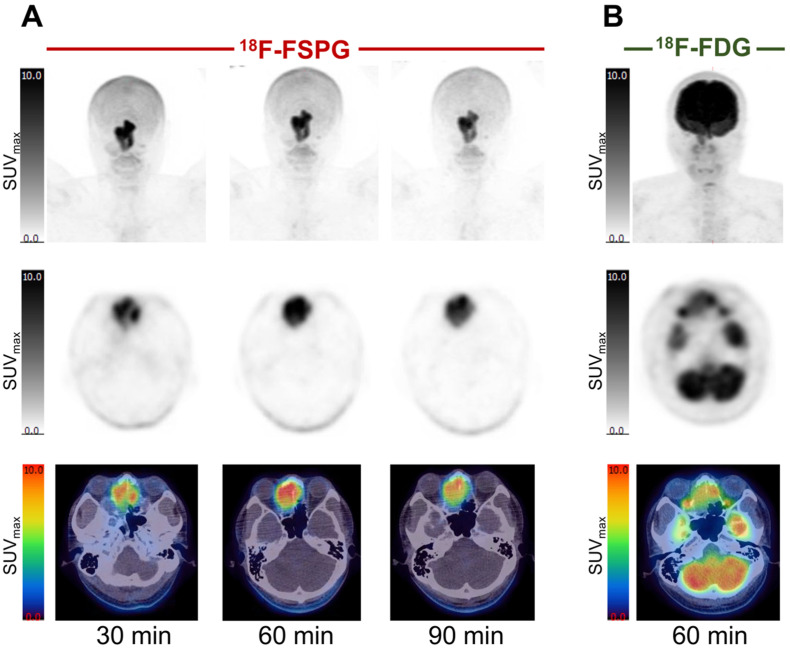
Comparison of [^18^F]FSPG PET/CT and [^18^F]FDG PET/CT imaging in a subject with HNSCC. (**A**) [^18^F]FSPG imaging at 30, 60 and 90 min p.i. From top to bottom: maximum intensity projection (MIP) scans, axial PET, and axial fused PET/CT images. (**B**) [^18^F]FDG PET/CT imaging (MIP, axial PET and axial fused PET/CT images) from the same subject at 60 min p.i.

In subjects with HNSCC, the retention of [^18^F]FSPG was not measurable at all time points due to low radioactivity levels in the tumor (Figure 4B). Even within this sample of five subjects, all with the same histology squamous cell carcinoma subtype, there was marked heterogeneity of [^18^F]FSPG retention among subjects, with high retention in one subject and low retention in the other four subjects. The subject with high [^18^F]FSPG retention (subject 11), only had imaging at 30, 60 and 90 min, limiting our assessment of earlier phase [^18^F]FSPG retention, but reached an SUV_max_ of 12.1 at 60 min p.i. This subject’s images are demonstrated in Figure 5, showing clear demarcation of the tumor in all three [^18^F]FSPG time course images, whereas high brain retention with [^18^F]FDG partially masked tumor visualization in the 60 min p.i. [^18^F]FDG PET/CT scan. Interestingly, although two of the subjects (12, 13) had previously received cancer treatment, their retention of [^18^F]FSPG was not notably different from the treatment-naïve subjects, with [^18^F]FDG confirming a viable tumor (SUV_max_ of 14.9 and 11.4, respectively). 

### 3.4. Inter-Lesion Variability across Metastases Is Apparent with [^18^F]FSPG 

**Figure 6:**
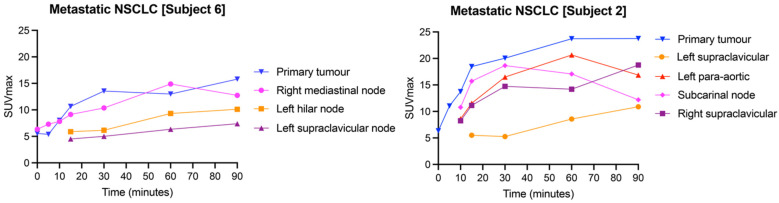
[^18^F]FSPG SUV_max_ time–activity curves of two different subjects (subjects 2 and 6) with metastatic NSCLC, demonstrating inter-subject and intra-subject variation in [^18^F]FSPG retention across primary tumors and metastases.

**Figure 7:**
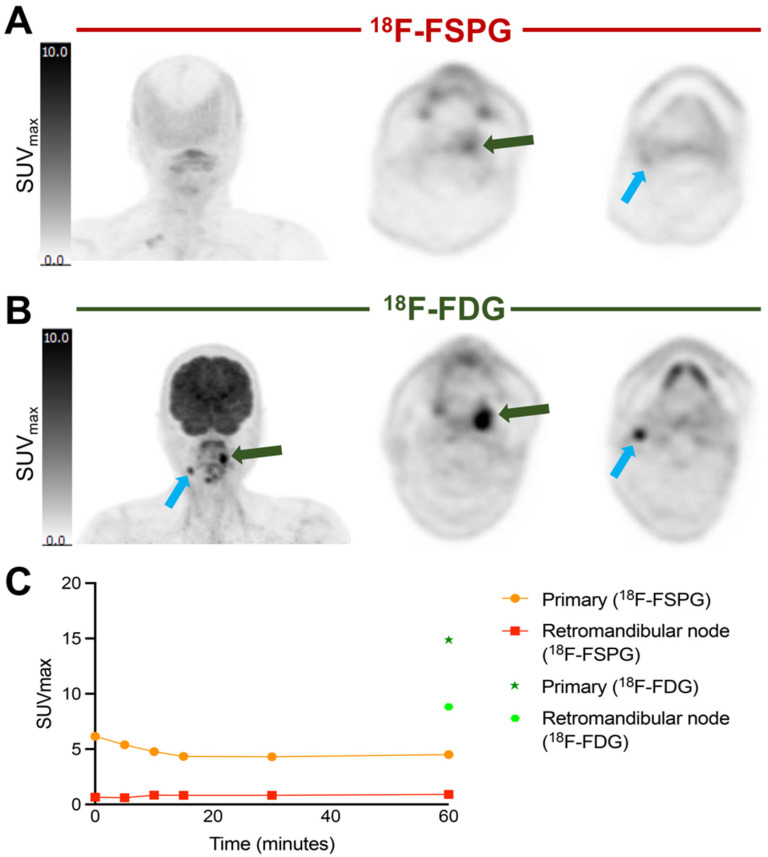
Comparison of [^18^F]FSPG vs. [^18^F]FDG imaging at 60 min p.i. of a primary HNSCC tumor (subject 12) and associated metastatic lesion. (**A**) [^18^F]FSPG imaging at 60 min p.i (coronal MIP, and axial views of the primary lesion within the left oropharynx (green arrow) and metastatic node (blue arrow)). (**B**) [^18^F]FDG imaging at 60 min p.i. (coronal MIP, and axial views of the primary and metastatic lesion). (**C**) Corresponding time–activity curves for both lesions.

Given the variability in [^18^F]FSPG retention among subjects, we next assessed whether radiotracer distribution was heterogenous across lesions in the same subject. In NSCLC subjects, the SUV_max_ of metastatic deposits was variable within and between subjects. All metastases >1 cm^3^ were included in the analysis, with a mean SUV_max_ of 7.8 ± 5.2 (range 0.8–23.4). Heterogeneity of [^18^F]FSPG retention between the primary lesion and metastases is illustrated in Figure 6, showing two subjects with NSCLC with multiple metastases. In subject 6, the three metastatic deposits had similar uptake kinetics to the primary tumor, with generally lower SUV_max_ values at each timepoint. In subject 2, the uptake kinetics of the four metastatic lesions varied from the primary tumor; for example, a subcarinal node had a maximal [^18^F]FSPG retention at 30 min p.i. (SUV_max_: 18.7), falling to 17.0 and 12.2 at 60 and 90 min p.i., respectively, whereas a supraclavicular node showed increasing [^18^F]FSPG retention up to 90 min p.i. (SUV_max_ of 5.3, 8.6 and 10.9 at 30, 60 and 90 min p.i., respectively). The median long axis for metastases was 21 mm (11–42 mm). Only two of the subjects with HNSCC had [^18^F]FDG-avid metastases, and only one of these had measurable [^18^F]FSPG retention. This subject had higher [^18^F]FDG than [^18^F]FSPG retention in both the primary tumor and the metastatic deposit, with an SUV_max_ of 14.9 ([^18^F]FDG) vs. 4.5 ([^18^F]FSPG) at 60 min p.i. in the primary, and an SUV_max_ of 8.8 ([^18^F]FDG) vs. 0.9 ([^18^F]FSPG) in the metastasis. These differences are highlighted in Figure 7, showing that the primary and metastatic deposits were clearly delineated with [^18^F]FDG, but were not avid for [^18^F]FSPG PET/CT.

### 3.5. Image Analysis Results in High Inter-Observer Concordance

Inter-observer reliability showed very high concordance. For the NSCLC cohort (170 data pairs), the *R*^2^ value was 0.92 (*p* < 0.01), with narrow confidence intervals of 0.94–0.97. In the HNSCC cohort (73 data pairs), the *R*^2^ value was 0.96 (*p* < 0.01), again with narrow confidence intervals of 0.97–0.99. Bland–Altman plots representing these data are illustrated in Supplementary Figure S2. 

## 4. Discussion

In this retrospective analysis of prospectively collected data, we compared [^18^F]FSPG PET/CT tumor retention in cancer subjects from different centers, finding heterogeneous inter-tumoral and inter-subject [^18^F]FSPG retention, despite consistent normal tissue biodistribution. [^18^F]FSPG has previously shown potential as a PET radiotracer for cancer detection, outperforming [^18^F]FDG in some cancer types, such as pancreatic [13] and hepatocellular [12] carcinomas, and could detect malignant lesions missed by standard-of-care imaging [3]. However, the variable retention characteristics of [^18^F]FSPG limits its utility as a general oncological diagnostic agent, likely underpinned by differences in the underlying tumor biology. 

Preclinical studies have shown that [^18^F]FSPG is specifically transported by the system x_c_^−^, cotransporter [18]. Cancer cells overexpress system x_c_^−^ [2,3], to support elevated GSH biosynthesis which maintains cellular redox homeostasis. System x_c_^−^ overexpression in tumors results in a concomitant increase in [^18^F]FSPG retention [19]. The differences in tumor biology highlighted by [^18^F]FSPG could be clinically exploited, as this may underpin a lesion-specific response to therapy. Some cancer cells can maintain a highly reduced intracellular environment to protect against treatment-induced oxidative stress, conferring therapy resistance [20,21]. Greenwood et al. [17] used matched drug-sensitive and drug-resistant ovarian cancer cells to show that resistance to chemotherapy corresponded with an increase in tumor antioxidant capacity. Drug-sensitive cells revealed high pre-treatment [^18^F]FSPG retention, whereas low intracellular [^18^F]FSPG retention was shown in the drug-resistant cells, indicating that pre-treatment [^18^F]FSPG imaging could highlight drug-resistant disease. To extrapolate this hypothesis to the findings in this cohort, subject 2 (NSCLC) exhibited high [^18^F]FSPG retention and would be hypothesized to have drug-sensitive cancer, whereas subjects 5 and 8 showed low retention and a higher probability of drug-resistance (Figure 4). With further clinical research, [^18^F]FSPG could act as a targeted PET radiotracer to predict response prior to therapy; this represents a significant unmet need in cancer care, as treatment resistance is responsible for up to 90% of cancer-related deaths [22,23,24]. 

Another unmet need in cancer care is early treatment-response assessment. For many cancers, [^18^F]FDG PET/CT imaging at 12 weeks is the standard-of-care for assessing treatment response [25,26]. Frequently, in those patients with poor response, this is too late for further intervention. In mouse models, a decrease in [^18^F]FSPG was observed both before glycolytic changes (as assessed with [^18^F]FDG) and tumor shrinkage [16,17]. In a further preclinical study, [^18^F]FSPG tumor retention was significantly decreased from baseline at only one week post-therapy, prior to changes in tumor volume, in tumors sensitive to therapy [27]. [^18^F]FSPG retention was static in treatment-resistant tumors, indicating no treatment response. As changes in [^18^F]FSPG preceded changes in standard-of-care methods of assessing treatment response, [^18^F]FSPG PET/CT could be used as an early marker of treatment-response assessment, enabling rapid transition to second-line treatment for those with treatment-resistant disease. A prospective clinical trial (NCT05889312) is planned for later in the year to assess [^18^F]FSPG as an early marker of response 2–8 weeks after commencement of (chemo)radiotherapy in NSCLC and HNSCC. This trial will set the scene for further interventional trials with the hope that imaging will identify patients that require radiotherapy dose-escalation, or prompt exchange to second-line therapy.

In the metastatic setting, it is important to assess response across the entire tumor burden. The advent of total-body PET [28] has now made it possible to scan a patient from head to foot in a matter of minutes, overcoming previous technical limitations. Our results show that even within subjects, lesions exhibit variable [^18^F]FSPG retention. For example, in NSCLC subject 6 the metastatic deposits exhibited similar [^18^F]FSPG retention characteristics to the primary tumor (Figure 6), predicting a consistent treatment response between lesions. Conversely, NSCLC subject 2 showed different [^18^F]FSPG retention characteristics between the primary tumor and metastases, and even between different metastases. We believe this to be a true finding; in both primary tumors and metastases we do not think the partial volume effect substantially contributed to the observed heterogeneity in activity, particularly when minimized with SUV_max_ measurements, with primary tumor median long-axis diameter of 28 mm (20–80 mm) and metastases 21 mm (11–42 mm). In patients exhibiting heterogeneous retention between lesions, a personalized approach to multimodality treatment may be required, with [^18^F]FSPG imaging used to monitor these patients on a lesion-by-lesion basis. 

Although there are now several human studies that have assessed [^18^F]FSPG in cancer patients, the wide range of cancer types and very small group sizes have limited interpretation and comparison between studies. Confounding factors can also influence the specificity of the [^18^F]FSPG signal: activated immune cells can overexpress system x_c_^−^ [29], and [^18^F]FSPG, like [^18^F]FDG, has been shown to exhibit high retention in a variety of inflammatory processes, such as sarcoid [30] and multiple sclerosis [31]. These factors may influence the specificity of [^18^F]FSPG signal, complicating image interpretation. Despite these limitations, our study has provided further insight into this biologically interesting radiotracer, allowing comparison of [^18^F]FSPG imaging across centers, and demonstrating high quality imaging with very high inter-observer concordance. We have also explored the heterogeneity of [^18^F]FSPG retention, and suggest why these differences occur and what potential they offer in the future. Prospective clinical studies are next required to assess the utility of [^18^F]FSPG in assessing early cancer-treatment response and treatment resistance.

## 5. Conclusions

[^18^F]FSPG PET/CT data between HNSCC and NSCLC and different centers revealed heterogeneity of radiotracer retention between tumor types, and between primary and metastatic tumors in individual subjects, despite similar normal organ kinetics. This heterogeneity limits the potential of [^18^F]FSPG as a general oncological diagnostic radiotracer. Variation in [^18^F]FSPG retention, however, suggests differences in underlying tumor biology. These differences could potentially be clinically exploited for the assessment of treatment resistance and early treatment response, which represent two of the most significant unmet cancer care needs worldwide.

## Figures and Tables

**Table 1:** Subject Demographics.

	Subject	Gender	Age	Pathology	Location	Prior Treatment
NSCLC subjects	1	Male	68	NSCLC, adenocarcinoma	Right lung	None
	2	Female	58	NSCLC, adenocarcinoma	Right lung	None
	3	Male	70	NSCLC, squamous cell carcinoma	Right lung	None
	4	Female	54	NSCLC, adenocarcinoma	Right lung	None
	5	Male	66	NSCLC, adenocarcinoma	Right lung	None
	6	Male	60	NSCLC, squamous cell carcinoma	Left lung	None
	7	Female	50	NSCLC, adenocarcinoma	Right lung	None
	8	Male	54	NSCLC, adenocarcinoma	Bilateral	None
	9	Male	62	NSCLC, adenocarcinoma	Right lung	None
	10	Male	55	NSCLC, adenocarcinoma	Right lung	None
HNSCC subjects	11	Male	44	HNSCC	Nasal cavity	None
	12	Male	73	HNSCC	Oropharynx/oral cavity (recurrent)	Chemoradiation, laryngectomy
	13	Male	71	HNSCC	Left maxillary sinus	Chemoradiation
	14	Male	74	HNSCC	Larynx	None
	15	Male	72	HNSCC	Nasopharynx	None

**Table 2:** Comparison of primary tumor SUV values with [^18^F]FDG and [^18^F]FSPG PET.CT imaging at 60 min p.i.

	Subject	Scan	SUV Max	SUV Mean	SUV SD	SUV Peak	MTV	SUV Max/Mean
NSCLC	1	[^18^F]FDG 60 min p.i.	11.8	7.1	1.7	8.8	3.1	1.7
		[^18^F]FSPG 60 min p.i.	7.3	4.6	1.3	6.4	4.5	1.6
	2	[^18^F]FDG 60 min p.i.	16.9	8.8	14.3	1.7	62.8	1.9
		[^18^F]FSPG 60 min p.i.	23.7	13.9	2.5	16.9	16.8	1.7
	3	[^18^F]FDG 60 min p.i.	11.8	7.0	1.5	9.7	34.8	1.7
		[^18^F]FSPG 60 min p.i.	11.4	6.2	1.5	8.8	13.1	1.8
	4	[^18^F]FDG 60 min p.i.	7.8	5.0	1.3	6.1	2.5	1.6
		[^18^F]FSPG 60 min p.i.	1.4	0.9	0.3	/	/	1.5
	5	[^18^F]FDG 60 min p.i.	15.1	9.9	2.9	12.4	2.9	1.5
		[^18^F]FSPG 60 min p.i.	1.6	1.1	0.3	/	/	1.5
	6	[^18^F]FDG 60 min p.i.	17.3	13.2	2.2	15.0	3.9	1.3
		[^18^F]FSPG 60 min p.i.	13.0	7.5	2.1	9.7	2.6	1.7
	7	[^18^F]FDG 60 min p.i.	10.3	6.8	1.5	8.9	10.4	1.5
		[^18^F]FSPG 60 min p.i.	4.3	2.6	0.9	/	/	1.6
	8	[^18^F]FDG 60 min p.i.	9.3	5.5	1.4	7.5	5.1	1.7
		[^18^F]FSPG 60 min p.i.	1.9	1.3	0.3	/	/	1.5
	9	[^18^F]FDG 60 min p.i.	12.6	6.7	2.3	7.7	2.0	1.9
		[^18^F]FSPG 60 min p.i.	12.3	7.8	2.1	/	1.4	1.6
	10	[^18^F]FDG 60 min p.i.	7.8	5.6	1.0	6.4	2.7	1.4
		[^18^F]FSPG 60 min p.i.	3.7	2.1	0.8	/	/	1.7
HNSCC	11	[^18^F]FDG 60 min p.i.	5.5	4.5	0.7	/	/	1.2
		[^18^F]FSPG 60 min p.i.	12.1	7.4	10.8	1.8	38.5	1.6
	12	[^18^F]FDG 60 min p.i.	14.9	8.5	2.2	10.8	3.7	0.6
		[^18^F]FSPG 60 min p.i.	4.5	4.1	0.5	/	/	0.9
	13	[^18^F]FDG 60 min p.i.	11.4	6.7	1.6	9.1	10.3	1.7
		[^18^F]FSPG 60 min p.i.	4.0	2.7	0.8	/	/	1.5
	14	[^18^F]FDG 60 min p.i.	Primary tumor not detectable with [^18^F]FDG					
		[^18^F]FSPG 60 min p.i.	6.3	4.1	1.1	/	/	1.5
	15	[^18^F]FDG 60 min p.i.	6.4	4.1	1.2	/	/	1.6
		[^18^F]FSPG 60 min p.i.	3.1	1.6	0.8	/	/	1.9

## Data Availability

Requests for access to imaging data should be made to the corresponding authors from which these data were obtained. For HNSCC, please contact Erik Mittra: mittra@ohsu.edu; for NSCLC, contact Dae Hyuk Moon: dhmoon@amc.seoul.kr.

## Supplementary Materials

The following supporting information can be downloaded at: https://www.mdpi.com/article/10.3390/cancers16071437/s1, Figure S1. Correlation of SUVmax at 60 mins p.i. of [^18^F]FPSG vs. [^18^F]FDG, in HNSCC primary tumors (A), NSCLC primary tumors (B) and NSCLC metastatic lesions (C). Only one patient with HNSCC had a metastatic deposit. Lines of linear regression and 95% confidence levels (broken lines) are shown, Figure S2. Analysis of inter-observer readings (SUVmax as measured by Observer 1 vs. Observer 2) demonstrated high correlation. All time points and patients were included. (A) NSCLC inter-observer correlation. Lines of linear regression and 95% confidence levels (broken lines) are shown. (B) Bland–Altman plots (difference vs average) of NSCLC. (C) HSNCC inter-observer correlation. Lines of linear regression and 95% confidence levels (broken lines) are shown. (D) Bland–Altman plot for HNSCC.

## Abbreviations

[^18^F]FDG	^18^F-2-fluoro-2-deoxy-D-glucose
[^18^F]FSPG	(4*S*)-4-(3-[^18^F]fluoropropyl)-L-glutamic acid
GSH	glutathione
PET	positron emission tomography
CT	computed tomography
HNSCC	head and neck squamous cell cancer
NSCLC	non-small-cell lung cancer
SUV	standardized uptake value
