# Supplementary material for: Clinical [18F]FSPG Positron Emission Tomography Imaging Reveals Heterogeneity in Tumor-Associated System xc− Activity
Source: Cancers (Basel). 2024 Apr 8;16(7):1437. doi: 10.3390/cancers16071437 (PMC11011143; doi:10.3390/cancers16071437)
Supplement: Supplementary file 1 [file cancers-16-01437-s001.zip › cancers-2890375-supplementary.pdf]

Supplementary data

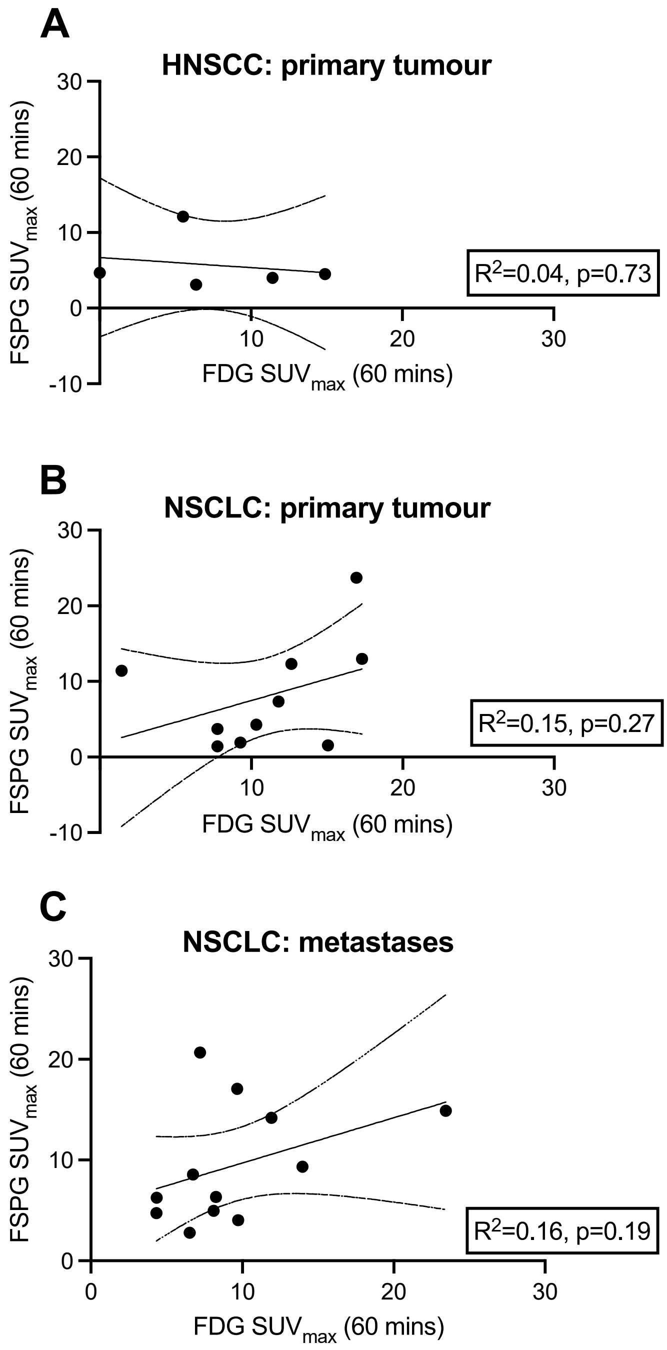

Supplementary Figure S1. Correlation of SUV_{max} at 60 mins p.i. of $[^{18}F]$FPSG vs. $[^{18}F]$FDG, in HNSCC primary tumors (**A**), NSCLC primary tumors (**B**) and NSCLC metastatic lesions (**C**). Only one patient with HNSCC had a metastatic deposit. Lines of linear regression and 95% confidence levels (broken lines) are shown.

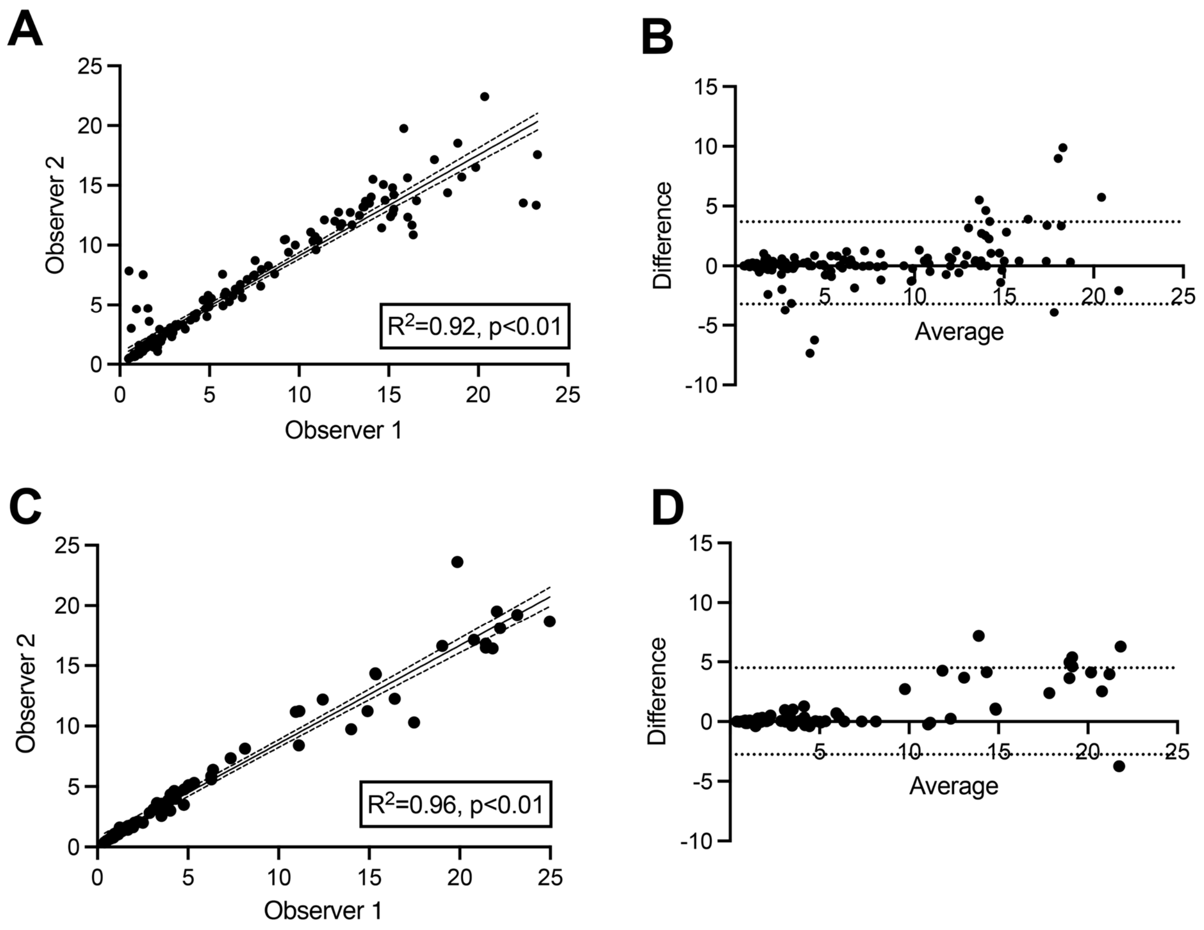

Supplementary Figure S2. Analysis of inter-observer readings (SUV_{max} as measured by Observer 1 vs Observer 2) demonstrated high correlation. All time points and patients were included. (**A**) NSCLC inter-observer correlation. Lines of linear regression and 95% confidence levels (broken lines) are shown. (**B**) Bland-Altman plots (difference vs average) of NSCLC. (**C**) HSNCC inter-observer correlation. Lines of linear regression and 95% confidence levels (broken lines) are shown. (**D**) Bland-Altman plot for HNSCC.
